# Analysis of Women’s Knowledge, Health Risk Perceptions, Beliefs and Avoidance Behaviour in Relation to Endocrine-Disrupting Chemicals in Personal Care and Household Products

**DOI:** 10.3390/toxics13050414

**Published:** 2025-05-21

**Authors:** Adrianna Trifunovski, Nooshin Khobzi Rotondi, Jennifer Abbass-Dick, Caroline Barakat

**Affiliations:** Faculty of Health Sciences, Ontario Tech University, 2000 Simcoe Street, Oshawa, ON L1G 0C5, Canada; nooshin.rotondi@ontariotechu.ca (N.K.R.); jennifer.abbassdick@ontariotechu.ca (J.A.-D.); caroline.barakat@ontariotechu.ca (C.B.)

**Keywords:** Health Belief Model, parabens, perceptions, knowledge, EDCs, personal care products, women’s health, Canada

## Abstract

Evidence highlights the association between endocrine-disrupting chemicals (EDCs) found in personal care and household products (PCHPs) and adverse reproductive and developmental health outcomes. Women are disproportionately at risk due to frequent use of PCHPs, encountering a variety of different chemicals daily. Despite known health risks, existing policies often fail to provide adequate protection, with gaps remaining in understanding women’s knowledge, risk perceptions, and beliefs about EDCSs in PCHP, as well as how these influence avoidance behaviours. This study examines women’s knowledge, health risk perceptions, beliefs, and avoidance behaviors regarding EDCs commonly found in PCHPs, including bisphenol A, lead, parabens, phthalates, perchloroethylene, and triclosan. Guided by the Health Belief Model, a questionnaire was administered to 200 women in the preconception and conception periods in Toronto, Canada. Analyses revealed that lead and parabens were the most recognized EDCs, while triclosan and perchloroethylene were the least known. Greater knowledge of lead, parabens, bisphenol A, and phthalates significantly predicted chemical avoidance in PCHPs. Higher risk perceptions of parabens and phthalates also predicted greater avoidance. Women with higher education and chemical sensitivities were more likely to avoid lead. These findings support the need for targeted education to improve awareness to reduce EDC exposure—especially among women.

## 1. Introduction

Individuals are increasingly exposed to harmful chemicals in everyday consumer products, which have become a significant part of daily life [1,2]. Among these, personal care and household products (PCHPs) are major sources of endocrine-disrupting chemicals (EDCs), with exposure occurring through dermal absorption, inhalation, or ingestion [3,4]. Women, as the primary users of PCHPs, may be particularly vulnerable to EDC exposure, encountering an estimated 168 different chemicals daily [5].

Personal care products include hygiene, grooming, and cosmetic items such as shampoo, toothpaste, and lotions [6], while household products like floor polish and dish soap serve cleaning functions [7]. Research indicates that individuals use at least two personal care products per day, averaging 11 applications within 24 h, totaling up to 44 applications daily, while dish, and stove cleaners are used daily, highlighting the frequent usage of a variety of PCHPs in a given day [3,6,7].

Manufacturers of PCHPs frequently add chemicals to enhance quality and shelf-life [4]. These additives—such as color enhancers, preservatives, plasticizers, antimicrobials, and solvents—often include EDCs like lead, parabens, bisphenol A (BPA), phthalates, triclosan, and perchloroethylene (PERC) [2,3,8].

Chronic exposure to these EDCs poses significant health risks (see Table 1) [9]. For instance, lead, used in cosmetics, disrupts hormonal functions and fetal development [10,11]. Parabens and phthalates, common in moisturizers, disinfectants, and air fresheners, are linked to reproductive toxicity and carcinogenic effects through estrogen mimicry [12,13]. BPA, found in plastic packaging, impairs reproduction and fetal growth [14]. Triclosan, present in antiseptics and soaps, is associated with miscarriage, infertility, and developmental toxicity [15,16]. PERC, used in dry cleaning and floor cleaners, is classified as a human carcinogen and reproductive toxicant [17,18].

### Study Scope and Objectives

Growing public concern in Canada has prompted calls for more transparent product labeling, particularly regarding broad terms such as “parfum” and “fragrance” [22]. These terms can hide the presence of dozens to hundreds of undisclosed chemical ingredients, many of which are known EDCs and fragrance allergens, even in PCHPs labelled “green” or “eco-friendly” [23]. The absence of full disclosures of ingredients poses significant challenges for consumers, who may unknowingly be exposed to EDCs, carcinogens, and other hazardous substances. Enhanced labeling standards are therefore critical to support informed decision-making and protect public health. However, it can take several years to test and form regulatory action [24]. Given this lag, it is imperative to increase awareness of these issues and of safer alternatives, such as chemical-free, vegan, and natural products [25]. Adopting safer alternatives has been shown to lower EDC exposure from PCHPs [26]; thus, understanding the factors that drive EDC avoidance is critical.

A recent study indicated a significant lack of awareness among women regarding the presence of harmful chemicals such as EDCs in PCHPs [27]. To highlight this, undergraduate female college students in California, USA were surveyed about their personal care product usage; 80% of women reported being unsure whether their products contained harmful chemicals, and 48.6% questioned their safety for daily use [28]. Research on pregnant women suggests a similar trend: while 60% recognize EDCs as health risks, those with higher education are more likely to engage in avoidance behaviors [29]. However, despite awareness of risks, only a small proportion intend to reduce cosmetic use during pregnancy [30]. Women who actively read product labels are more likely to mitigate exposure [31]. However, among reproductive-aged women aware of risks, only 29% adopt avoidance behaviors, highlighting a gap between awareness and action [32].

Research gaps remain regarding women’s access to product safety resources and their health risk perceptions of hazardous EDCs in PCHPs, along with their associated health risks, beliefs, and avoidance behaviors. To address the gaps, this study aimed to achieve the following: (i) assess women’s knowledge (access to resources), health risk perceptions, beliefs, and avoidance behavior related to EDC exposure in PCHPs; and (ii) examine associations between demographic factors and knowledge (access to resources), health risk perceptions, beliefs, and avoidance behaviour concerning EDC exposure in PCHPs. To achieve these objectives, the Health Belief Model (HBM) was used. The HBM aims to explain behavior change by evaluating an individual’s motivation and perceived ability to adopt healthier behaviors. For instance, a woman who perceives a heightened risk of breast cancer due to paraben exposure and understands its health implications may become more concerned about chemical-based PCHPs. If she believes that choosing paraben-free products can lower her risk, she is more likely to adjust her purchasing behavior accordingly.

## 2. Methods

### 2.1. Study Design 

This study utilized a researcher-designed questionnaire based on the HBM. The questionnaire underwent pilot testing, and preliminary analyses indicated an acceptable reliability of participants’ responses and constructs measured using Cronbach’s alpha [33]. The questionnaire was subsequently used to assess women’s knowledge, health risk perceptions, beliefs, and avoidance behaviors regarding EDCs in PCHPs.

The questionnaire began by collecting information about demographic characteristics, followed by dedicated sections for each EDC: lead, parabens, phthalates, BPA, triclosan, and PERC. Within each section, four scales measured participants’ knowledge, health risk perceptions, beliefs, and avoidance behaviors regarding each EDC. Beliefs were measured using five items assessing participants’ views on the health impacts of each EDC. Health risk perceptions were measured with seven items evaluating the perceived health risks associated with EDC exposure. Knowledge was assessed through six items examining access to information, perceived sufficiency of product safety knowledge, and interest in further information. Avoidance behavior was measured with six items focusing on purchasing practices related to avoiding EDCs in PCHPs. Items utilized a six-point Likert scale (ranging from Strongly Agree to Strongly Disagree), with the exception of avoidance behavior, which used a five-point scale (from Always to Never). 

### 2.2. Sample

The study focused on women aged 18 to 35, a range selected to capture the pre-conception and conception stages. According to Statistics Canada, women typically have their first child between the ages of 25 and 35, with an average age of 32 [34]. Furthermore, the study population was selected based on the higher usage of PCHPs by women, with the recognition that their exposure to EDCs from PCHPs may have implications for prenatal and postnatal exposures in infants. To meet the inclusion criteria, participants must have identified as female (sex at birth) and been able to read and write in English. The exclusion criteria were those identifying their sex as male at birth, and those who were outside of the 18–35 age range. Ethical approval for the study was granted by the Ontario Tech University Ethics Committee [REB#16949].

### 2.3. Data Collection

The questionnaire was distributed to participants in person and online via a Google Forms link. Most of the study participants (90%) were recruited in-person at the 2022 Toronto National Women’s Show (Toronto, Canada, 18–19 November 2022) and Ontario Tech University (9 February 2023). The research team members were readily available to help participants with filling out the questionnaire, as well as engaging in conversations with respondents on their behaviours and selection of PCHPs. Additionally, some of these participants were invited to participate in a semi-structured interview about their decision-making around PCHPs.

For the final recruitment method, an online promotional campaign was launched across various social media platforms (such as Instagram and LinkedIn) between December 2022 and January 2023. Participants were recruited voluntarily and provided written informed consent after reviewing the study details, ensuring their understanding of the research procedures. All data collected were anonymized to protect participant confidentiality.

### 2.4. Data Analysis

Data analysis was conducted using SPSS v.28. All data was cleaned and coded prior to analysis. The coding system used was similar to those from previous studies, which was the utilization of a Likert-scale coded in a positive direction [35,36]. Mean imputation was used to fill in ‘unsure’ responses. If the respondent answered ‘unsure’, their response was imputed using the average of their other responses in that scale [37]. All of the surveys were used (*n* = 200) for the interpretation of results.

#### 2.4.1. Calculating Respondent Scores

An index was created for knowledge (score range: 6–30), health risk perceptions (score range: 7–35), beliefs (score range: 5–25), and avoidance behavior (score range: 6–30) by summing the Likert-scale responses for each participant, resulting in four distinct composite scores for each of the six EDCs [35,38]. Descriptive statistics, including mean, standard deviation, and median, were used to summarize respondent scores and assess the central tendency and variation for each index. Participants who were aware of a given EDC had four corresponding composite scores for that chemical. A higher score resulted in a higher level of understanding for each construct.

#### 2.4.2. Examining Respondent Scores and Associations with Demographic Variables

This study analyzed associations between knowledge, health risk perceptions, beliefs, and avoidance behavior as outcome variables in relation to demographic factors. Since the outcome variables exhibited a non-normal distribution, non-parametric statistical tests were used [39]. The Mann–Whitney U test was applied for comparisons involving two subgroups, while the Kruskal–Wallis H test was used for sociodemographic variables with more than two subgroups. Due to low respondent awareness of triclosan and PERC, the analysis focused on lead, parabens, BPA, and phthalates.

Demographic variables included age, ethnicity, annual household income, education level, and household chemical sensitivity. Age was categorized as ‘early adulthood’ (18–25 years) and ‘later adulthood’ (26–35 years). Ethnicity was classified as non-white or white. The responses were used to dichotomize education into ‘post-secondary completion’ versus ‘non-completion’ due to the high proportion of secondary education in the sample. Household income was categorized as above or below the Low-Income Cut-Off (LICO), adjusted for household size and pre-tax income within the Census Metropolitan Area [40]. Chemical sensitivity was grouped into three categories: diagnosed in oneself or a household member, don’t know, or no diagnosis.

#### 2.4.3. Estimates Associated with EDC Avoidance in PCHP

Multivariable ordinal regression assessed the relationship between avoidance behavior (dependent variable) toward EDCs in PCHPs and knowledge, health risk perceptions, beliefs, age, ethnicity, education, household income, and self-reported chemical sensitivity (independent variables). Multicollinearity was evaluated using the variance inflation factor (VIF) to ensure variable validity [41,42].

Avoidance behavior, the primary outcome, was analyzed across four ordinal variables for lead, parabens, BPA, and phthalates. The avoidance behavior index (range: 6–30) was categorized using quartile-based cutoffs [43]. Lead, parabens, and phthalates each had four categories, while BPA had three, reflecting a 75th percentile score of 30, with 25% of participants exhibiting maximum avoidance for BPA in PCHPs.

## 3. Results

### 3.1. Sample Characteristics

The sample characteristics can be found in Table 2. Of the 200 participants, 57% (*n* = 114) were aged 18–25, and 38% (*n* = 76) were 26–35. In terms of ethnicity, 58% (*n* = 134) identified as non-white, and 42% (*n* = 84) were white. Regarding annual household income (CAD$), 65% (*n* = 131) had incomes above the LICO, and 21% (*n* = 42) had incomes equal to or below the LICO. The sample was highly educated, with 66% (*n* = 133) holding post-secondary degrees or higher, and 29% (*n* = 58) only completing high school. Most respondents (79%) were born in Canada, while 19% (*n* = 38) were born outside of Canada. Additionally, 10% (*n* = 20) reported diagnosed chemical sensitivities. A small subset (*n* = 3) were pregnant, 5% *(n* = 9) were attempting pregnancy, and 13% (*n* = 26) had a child under 18. Statistical tests found no significant differences between recruitment sites, allowing for data pooling into a single sample.

### 3.2. Respondent Scores

**Figure 1:**
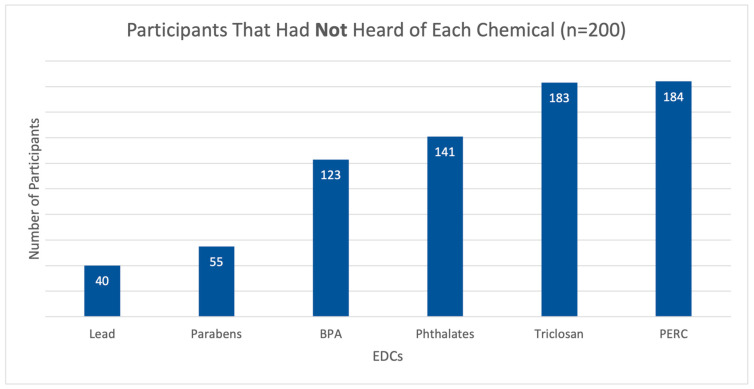
Proportion of participants who had never heard of lead, parabens, BPA, phthalates, triclosan, and PER.

Figure 1 illustrates the distribution of participant responses indicating that they had ‘never heard’ of specific EDCs of interest. The chemicals with the highest proportion of participants reporting lack of familiarity were PERC (*n* = 184), triclosan (*n* = 183), phthalates (*n* = 141), and BPA (*n* = 123). A comparatively lower number of participants indicated unfamiliarity with parabens (*n* = 55) and lead (*n* = 40). The results of the respondent scores from all indices are presented in Table 3. From this analysis, it was found that health risk perception scores for parabens displayed the lowest scores for all dimensions out of all EDCs. Triclosan and PERC displayed the highest scores across all indices.

### 3.3. Respondent Scores and Associations with Demographic Variables

Bivariate analysis identified six statistically significant associations between ethnicity, income, chemical sensitivity diagnosis, and household income for lead, parabens, BPA, and phthalates (Table 4). A statistically significant association was observed between education level and the knowledge of lead. Participants without post-secondary education had a higher mean rank (87.5) compared to those with post-secondary education (71.5) (Table 3). Education level was also significantly associated with the knowledge of parabens. Participants without post-secondary education had a higher mean rank (81.0) than those with post-secondary education (63.5).

A significant association was found between education level and belief in the harm of parabens. Participants without post-secondary education had a higher mean rank (78.0) than those with post-secondary education (62.3). A significant association was observed between chemical sensitivity diagnosis and health risk perceptions of lead. Participants with a diagnosed chemical sensitivity (or a household member with a diagnosis) had a higher mean rank (100.1) compared to those who were unsure or did not report a diagnosis. Ethnicity was significantly associated with health risk perceptions of BPA. For instance, white participants had a higher mean rank (39.7) than non-white participants (28.6).

A statistically significant association was found between household income and avoidance behavior toward parabens. Participants with incomes at or below the LICO had a higher mean rank (78.6) compared to those above the LICO (59.6). Household income was also significantly associated with avoidance behavior toward phthalates. Participants with incomes at or below the LICO had a higher mean rank (33.6) than those above the LICO (23.6).

Table 5 presents the results of the ordinal regression analysis, identifying nine significant associations between demographic and physical variables, knowledge, health risk perceptions, beliefs, and avoidance of EDCs in PCHPs. A multicollinearity check confirmed no collinearity between independent variables prior to analysis.

Knowledge was a significant predictor of avoidance behavior across all models, with positive associations observed for lead (OR = 1.41, 95% CI [1.25, 1.62], *p* < 0.001), parabens (OR = 1.26, 95% CI [1.10, 1.44], *p* < 0.001), BPA (OR = 1.73, 95% CI [1.25, 2.38], *p* < 0.001), and phthalates (OR = 1.57, 95% CI [1.18, 2.08], *p* < 0.001). Health risk perceptions were also a significant factor related to an increased avoidance of parabens (OR = 1.17, 95% CI [1.05, 1.30], *p* < 0.01) and phthalates (OR = 1.26, 95% CI [1.01, 1.57], *p* < 0.05).

Beliefs toward the harm of parabens demonstrated a significant positive relationship with avoidance behavior (OR = 1.14, 95% CI [1.02, 1.28], *p* < 0.05). Participants with a self-reported diagnosis of chemical sensitivity were significantly more likely to avoid lead (OR = 5.86, 95% CI [1.62, 21.1], *p* < 0.01) compared to those without a diagnosis or those unsure of having a diagnosis. Conversely, not completing post-secondary education was significantly associated with lower avoidance of lead (OR = 0.21, 95% CI [0.09, 0.53], *p* < 0.001) compared to individuals who completed post-secondary education.

## 4. Discussion

EDCs such as lead, parabens, BPA, phthalates, triclosan, and PERC are pervasive in consumer products, yet a considerable portion of women remain uninformed about them. In this study, it was uncovered that the majority of participants, specifically 94%, were unfamiliar with PERC, followed by 93% who had never heard of triclosan. Despite PERC and triclosan being the least recognized, they attained the highest mean scores in knowledge, health risk perceptions, beliefs, and avoidance behavior when compared to all six EDCs. This could potentially be attributed to participants having engaged in comprehensive research on multiple chemical ingredients, thereby fostering a predisposition toward adopting avoidance concerning chemical exposures stemming from PCHPs [31].

The awareness of EDCs varied among participants. Only 29% had heard of phthalates, lower than the 44% reported in previous studies [32]. BPA awareness was also low, with 38% awareness in comparison to 93% in previous studies [32]. In contrast, 72% were familiar with parabens, higher than the 24% reported by a previous study [31]. Despite significant media attention in the early 2010s, BPA recognition was relatively low in our study [44]. This discrepancy may be explained by the younger age of our sample (18–25 years), as they may have had less exposure to earlier public discussions on BPA. Furthermore, parabens have been receiving greater attention in mainstream media due to health concerns, prompting a shift toward “paraben-free” product labels [45]. This may explain the higher recognition of parabens in our study compared to previous ones.

Among the six EDCs analyzed, lead had the lowest mean avoidance behavior score in PCHPs despite being the most widely recognized by participants. This discrepancy suggests that while awareness of lead is high—likely due to extensive historical media coverage and public health campaigns following its 2012 ban in Canadian consumer products [46]—this recognition does not necessarily translate into active avoidance behaviors. One possible explanation is that the ban may have led participants to perceive lead as a resolved risk, reducing the urgency to seek or avoid products containing it. This pattern aligns with the HBM, which states that perceived susceptibility and severity—along with cues to action—are key drivers of behavior change. In the absence of current or perceived personal risk, participants may not actively avoid lead-containing PCHPs, assuming regulatory action has already eliminated the threat. The avoidance of lead in PCHPs was significantly associated with knowledge, post-secondary education, and self-reported household chemical sensitivity. Participants with post-secondary education were more likely to avoid lead than those without, aligning with prior research showing that relatively highly educated women adopt more behaviors to reduce chemical exposure [29,32,47]. Additionally, individuals with diagnosed chemical sensitivity—either in themselves or a household member—were more likely to avoid lead. Those with chemical sensitivities often experience physical symptoms from exposure and may exhibit heightened chemical awareness and avoidance behaviors [48]. Notably, 10% of participants in this study reported a chemical sensitivity, a higher prevalence than in previous studies [49,50]. This increase may be because the questionnaire asked for an MCS diagnosis of the entire household, as opposed to previous studies where only the individual participant was asked.

Participants scoring higher on the knowledge scale were found to be significantly and positively associated with avoiding lead, parabens, BPA, and phthalates in their PCHPs. This may suggest that knowledge can aid in helping women choose healthier PCHP alternatives. This finding is aligned with recent findings, where women who had more knowledge of the danger products posed to their health with long-term use were more likely to purchase alternative products [31,51]. Furthermore, a participant scoring higher on the health risk perception scale was positively associated with avoiding parabens and phthalates in their PCHPs, and a higher belief score emerged as an estimate in avoiding parabens in PCHPs. Overall, these findings suggest that higher health risk perceptions and beliefs toward the harmful effects of EDCs may result in a higher likelihood of avoiding PCHPs containing EDCs, in line with previous findings [32,52].

The results of this study support and validate the HBM, the guiding theoretical framework for this research. According to the HBM, individuals are more likely to engage in preventive behaviors—such as avoiding EDCs in PCHPs—when they possess adequate knowledge, perceive a high level of risk, and hold strong beliefs about the potential health consequences of exposure [53]. In our study, participants with higher composite scores in knowledge, health risk perceptions, and beliefs also reported higher avoidance behaviors, aligning with the HBM’s core constructs. Importantly, they also highlight how targeted public health interventions that enhance knowledge, clarify personal risk, and strengthen belief in the effectiveness of avoidance strategies may effectively motivate consumers to choose EDC-free PCHPs [54]. Future campaigns might benefit from integrating HBM-informed messaging to encourage more widespread adoption of safer product choices [55].

A key limitation of this study is that the sampling method led to a sample that was predominantly highly educated and of higher socioeconomic status, potentially underrepresenting lower-income populations. Other limitations include sample size, recall bias, and response bias. Despite these limitations, this work adds to the under-researched area of women’s knowledge, health risk perceptions, beliefs, and avoidance behaviours relating to EDCs commonly found in PCHPs. The study results emphasize the importance of education and the distribution of knowledge to this group of women so that they can better protect themselves, their families, and a potentially growing fetus from harmful chemicals with endocrine-disrupting potential.

For future research, we recommend using a larger sample size than 200 as well as a wider age interval to capture more of the population. Future research may benefit from including men as well as gender-diverse groups in analysis to further understand the health perspectives of these groups, as well as sampling in different geographical locations, as this may affect the results and recognition of different EDCs [27].

## Figures and Tables

**Table 1:** Roles, sources, and health impacts of commonly found EDCs in PCHPs.

EDC	Sources	Functions	Health Impacts	References
Lead	- Cosmetics (lipsticks, eyeliner) - Household cleaners	Colour enhancer	- Infertility - Menstrual disorders - Disturbances to fetal development - Possibly carcinogenic (IARC: Group 2A)	[9,10,11,18,19]
Parabens	- Shampoos & conditioners - Lotions - Cosmetics (foundation) - Antiperspirants - Household cleaners (disinfectants, air fresheners)	Preservative	- Carcinogenic potential - Estrogen mimicking/hormonal imbalances - Reproductive effects/impaired fertility	[2,3,12,13,20]
Bisphenol A (BPA)	- Plastic packaging of PCHP - Antiperspirants - Detergents - Conditioners - Lotions - Soaps	Plasticizer	- Fetal disruptions/placental abnormalities - Reproductive effects	[3,13,14,20]
Phthalates	- Scented PCHP - Hair care products - Lotions - Cosmetics - Antiperspirants - Household cleaners (disinfectants, air fresheners)	Preservative, plasticizer	- Estrogen mimicking/hormonal imbalances - Reproductive effects/impaired fertility	[2,3,12,13,20]
Triclosan	- Toothpaste/mouth wash - Body washes - Dish soaps - Bathroom cleaners - Antiperspirants	Antimicrobial	- Miscarriage - Impaired fertility - Fetal developmental effects	[3,8,15,16,20]
Perchloroethylene (PERC)	- Spot removers - Floor cleaners - Furniture cleaners - Dry cleaning		- Probable carcinogen (IARC; Group 2A) - Reproductive effects/impaired fertility	[17,19,21]

**Table 2:** Sociodemographic characteristics of the study sample (*n* = 200).

Demographic Variables	n%
Age	
18–25	114 (57)
26–35	76 (38)
Prefer not to say	10 (5)
Education	
High school completed	58 (29)
Post-secondary completed	133 (66)
Prefer not to say	9 (5)
Annual Household Income	
Below or equal to LICO	42 (21)
Above LICO	131 (65)
Prefer not to say	27 (13)
Ethnicity	
Non-white	134 (58)
White	84 (42)
Birthplace	
Canada	157 (79)
Other	38 (19)
Prefer not to say	5 (2)
Diagnosed with chemical sensitivity	
Yes	20 (10)
No	153 (76)
Don’t know	22 (11)
Prefer not to say	5 (3)
Pregnant	
Yes	3 (1.5)
No	193 (96.5)
Prefer not to say	1 (0.5)
Attempting pregnancy	
Yes	9 (5)
No	183 (92)
Prefer not to say	2 (1)
Parent to a child under the age of 18 years old	
Yes	26 (13)
No	171 (86)
Prefer not to say	3 (1)

**Table 3:** Respondent scores of indices related to knowledge, risk perceptions, beliefs, and avoidance behavior for each EDC [33].

Toxin	Mean (SD); Median			
	Health Risk Perceptions (7–35)	Beliefs (5–25)	Knowledge (Access to Resources) (6–30)	Avoidance Behaviour (6–30)
**Lead (*n* = 161)**	29 (4); 28	19 (4); 20	23 (4); 23	19 (7); 18
**Parabens (*n* = 142)**	27 (5); 28	17 (4); 17	23 (4); 23	21 (6); 21
**BPA (*n* = 76)**	29 (5); 29	20 (3); 20	24 (4); 24	23 (6); 24
**Phthalates (*n* = 55)**	28 (5); 28	18 (4); 18	23 (4); 24	22 (6); 22
**Triclosan (*n* = 14)**	31 (6); 32	22 (3); 23	26 (4); 25	24 (8); 30
**PERC (*n* = 12)**	32 (4); 35	22 (4); 23	25 (5); 25	23 (6); 24

**Table 4:** Bivariate results on relationships between age, ethnicity, education, income, MCS and indices related to knowledge, health risk perceptions, beliefs, and avoidance behaviour for four EDCs.

Covariates	EDCs															
	Lead (*n* = 160)				Parabens (*n* = 145)				BPA (*n* = 72)				Phthalates (*n* = 55)			
	Mean Rank				Mean Rank				Mean Rank				Mean Rank			
	K	RP	B	AB	K	RP	B	AB	K	RP	B	AB	K	RP	B	AB
Age (years)																
18–25	75.5	70.2	72.6	74.4	62.7	63.3	64.7	65.9	31.3	31.2	32.7	33.2	24.0	28.5	28.5	26.7
26–35	78.2	81.3	78.8	82.6	72.8	68.3	66.5	70.8	39.8	35.1	36.7	39.6	30.3	25.3	25.3	27.4
Ethnicity																
Non-white	74.9	76.9	79.3	80.0	71.9	64.2	71.7	69.8	32.1	28.6	33.3	34.2	28.2	29.0	27.6	29.6
White	76.2	69.5	68.7	72.4	66.6	72.5	63.4	71.3	40.0	**39.7** *	37.0	38.4	26.7	25.6	27.3	24.9
Highest educational attainment																
No-post secondary	**87.5** *	74.4	77.3.	75.2	**81.0** *	74.3	**78.0** *	75.8	33.6	31.1	31.7	35.0	32.6	30.9.	34.0.	31.2
Post-Secondary	71.5	73.8	73.4.	77.7	63.5	63.8	62.3	66.6	34.1	32.3	34.0	35.0	25.2.	25.8.	24.8.	25.6
Annual household income (CAD)																
Equal/below LICO	68.5	65.6	72.9	76.0	73.9	71.7	68.3	**78.6** *	34.6	35.5	33.8	39.0	30.4	31.6	32.5	**33.6** *
Above LICO	66.5	66.1	72.6	65.3	60.0	59.3	60.2	59.6	30.1	28.6	30.3	30.6	24.9	24.5	24.1	23.6

* *p* < 0.05; Abbreviations: K = Knowledge, RP = Risk perceptions, B = Beliefs, AB = Avoidance behaviour; Mann–Whitney U Test used for age, ethnicity, highest educational attainment, annual household income, and chemical sensitivity diagnosis.

**Table 5:** Ordinal regression model of avoidance behaviour for lead, parabens, phthalates, and BPA.

	Lead	Parabens	BPA	Phthalates
	*n* = 124	*n* = 113	*n* = 56	*n* = 47
Categories	OR (95% CI)	OR (95% CI)	OR (95% CI)	OR (95% CI)
Risk Perceptions	1.08 (0.97, 1.21)	1.17 ** (1.05, 1.30)	1.08 (0.83, 1.39)	1.26 * (1.01, 1.57)
Knowledge				
(Access to resources)	1.41 *** (1.25, 1.62)	1.26 *** (1.10, 1.44)	1.73 *** (1.26, 2.38)	1.57 ** (1.18, 2.08)
Belief	0.99 (0.88, 1.11)	1.14 * (1.02, 1.28)	1.09 (0.81, 1.46)	1.20 (0.95, 1.52)
Age				
18–25				
26–35	0.58 (0.26, 1.29)	1.04 (0.46, 2.34)	0.35 (0.07, 1.76)	2.88 (0.52, 15.8)
Ethnicity				
(Non-white)				
White	0.50 (0.24, 1.04)	1.19 (0.56, 2.52)	0.63 (0.12, 3.31)	0.48 (0.11, 2.05)
Education				
(Post-secondary)				
No Post-secondary	0.21 *** (0.09, 0.53)	0.59 (0.23, 1.51)	0.83 (0.16, 4.36)	0.42 (0.07, 2.43)
Income				
(Above LICO)				
Below LICO	1.81 (0.74, 4.38)	2.16 (0.85, 5.48)	2.16 (0.27, 17.5)	2.77 (0.51, 15.0)
Chemical Sensitivity Diagnosis				
(No Diagnosis)				
Self-reported				
Diagnosis	5.86 ** (1.62, 21.1)	3.33 (0.97, 11.4)	1.57 (0.17, 14.5)	1.63 (0.25, 10.8)
Do not know	1.19 (0.38, 3.72)	1.37 (0.41, 4.61)	5.24 (0.33, 82.8)	0.12 (0.09, 1.73)
Model Fit				
Model fit				
information	<0.001	<0.001	<0.001	<0.001
Pearson				
Goodness of fit	0.01	0.884	0.04	0.673
McFadden				
Pseudo-R square	0.227	0.221	0.45	0.478
Test of parallel lines	0.056	0.105	0.679	0.998

* *p* < 0.05. ** *p* < 0.01. *** *p* < 0.001.

## Data Availability

The data that support the findings of this study are available from the corresponding author, Adrianna Trifunovski, upon request.
